# Impact of a mobile health intervention based on multi-theory model of health behavior change on self-management in patients with differentiated thyroid cancer: protocol for a randomized controlled trial

**DOI:** 10.3389/fpubh.2024.1327442

**Published:** 2024-01-11

**Authors:** Yang Jiang, Xiangju Sun, Maomin Jiang, Hewei Min, Jing Wang, Xinghua Fu, Jiale Qi, Zhenjie Yu, Xiaomei Zhu, Yibo Wu

**Affiliations:** ^1^Jitang College, North China University of Science and Technology, Tangshan, China; ^2^Clinical Pharmacy, The Fourth Affiliated Hospital of Harbin Medical University, Harbin, China; ^3^School of Public Affairs, Xiamen University, Xiamen, China; ^4^School of Public Health, Peking University, Beijing, China; ^5^The Fourth School of Clinical Medicine, Harbin Medical University, Harbin, China; ^6^School of Journalism and Communication, Zhengzhou University, Zhengzhou, China; ^7^School of Nursing, Tianjin Medical University, Tianjin, China; ^8^Department of Pharmacy, Beidahuang Group General Hospital, Harbin, China

**Keywords:** differentiated thyroid cancer, mHealth, MTM, health education, self-management

## Abstract

**Introduction:**

Theoretical models of health behavior are important guides for disease prevention and detection, treatment and rehabilitation, and promotion and maintenance of physical and mental health, but there are no intervention studies related to differentiated thyroid cancer (DTC) that use theoretical models of health as a guide. In this study, we used a microblogging platform as an intervention vehicle and mobile patient-doctor interactive health education as a means of intervention, with the aim of improving the health behaviors of DTC patients as well as the corresponding clinical outcomes.

**Methods:**

This research project is a quantitative methodological study, and the trial will be a single-blind, single-center randomized controlled trial conducted at the Fourth Hospital of Harbin Medical University in Harbin, Heilongjiang Province. The study subjects are patients over 18 years of age with differentiated thyroid cancer who were given radioactive iodine-131 therapy as well as endocrine therapy after radical surgery for thyroid cancer. The intervention group will receive MTM-mhealth, and the realization of health education will rely on the smart terminal WeChat platform. Routine discharge education will be given to the control group at discharge. The primary outcome will be change in thyroid-stimulating hormone (TSH) from baseline and at 3 and 6 months of follow-up, and secondary outcomes will include change in self-management behavior, social cognitive and psychological, and metabolic control.

**Discussion:**

This study will explore a feasible mHealth intervention program applied to a population of DTC patients using the Multi-theory model of health behavior change (MTM) as a guide, with the aim of evaluating the MTM-based intervention program for clinical outcome improvement in DTC patients, as well as determining the effectiveness of the MTM-based intervention program in improving self-management skills in DTC patients. The results of this study will indicate the feasibility as well as the effectiveness of the application of health theoretical modeling combined with mHealth applications in disease prognostic health management models, and provide policy recommendations and technological translations for the development of mobility-based health management applications in the field of health management.

## 1 Introduction

Thyroid cancer can be divided into differentiated thyroid cancer and undifferentiated thyroid cancer according to tissue classification, in which differentiated thyroid cancer (DTC) develops in more than 90% of patients (1, 2), and the global incidence of thyroid cancer increased by about 20% from 1990 to 2013 (3). According to the Annual Report of China Tumor Registry, the incidence of thyroid cancer in China has continued to increase from 2008 to 2018, and the incidence is likely to continue to rise in the foreseeable future (4). Wiltshire et al. conducted a systematic review of studies in Europe, North America, Asia, Oceania, and South America, confirming that the overall incidence of thyroid cancer has continued to increase globally (5).

Surgical resection is the standard of care for most patients with DTC. In particular, low-risk patients with highly differentiated thyroid cancer can be treated with surgical resection only, without the need for excessive radiation therapy (6–8), whereas patients with high-risk features require thyroid-stimulating hormone (TSH) suppression and radioactive iodine therapy (RAI) in conjunction with each other (9, 10).DTC is a TSH-dependent tumor, and TSH is able to stimulate the expression of TSH receptors in normal thyroid cells and DTC cells, leading to abnormal proliferation of normal thyroid tissue and cancerous tissue that may remain, increasing the likelihood of recurrence (11–13). Therefore, TSH suppression therapy plays a crucial role in postoperative DTC, particularly for patients undergoing total or near-total resection. These patients require thyroid hormone supplementation to lower TSH levels after surgery, not only to compensate for hormone deficiencies but also to impede the growth of DTC cells and prevent recurrence (14–17). Consequently, the continuous self-management of DTC patients, guided by medical professionals, becomes pivotal for effective TSH suppression therapy. Unfortunately, the existing postoperative care measures are insufficient, leading to various disease-specific health issues following initial treatment (18–20). Thus, it is imperative to adopt more innovative and effective approaches to enhance the prevention and treatment of differentiated thyroid cancer, ultimately improving prognosis and reducing the risk of recurrence.

With the advancement of medical technology, there is an increasing demand for personalized and precise disease management in patients with thyroid tumors. Studies have shown that fatigue and insomnia are two of the most common symptoms in DTC patients, for whom self-monitoring of serum TSH levels should be proactive in order to achieve a favorable prognosis (21, 22). The definition of self-management is for patients to take on the task of managing their own health (9, 23). This self-management by patients by rationalizing their exercise, diet, routine and taking medication in a self-disciplined manner is one way to address this need and may have a crucial role in improving the cure rate of patients with differentiated thyroid cancer.

The development of mobile technology has led to the gradual popularization of social software such as WeChat, where communication between patients and physicians can be shifted from offline to online, in addition, the World Health Organization has called for the expansion of the use of digital technology to improve health support for patients, which have effectively contributed to the development of mobile health (mHealth) (24–28). As a result, mHealth is widely used by a large number of healthcare entities and plays an important role in the prevention and treatment of chronic diseases such as hypertension, diabetes, asthma, and postoperative rehabilitation of the heart and lungs and can save up to seven billion dollars in budgets annually (29, 30). The use of cell phones and mHealth apps continues to proliferate, with an increasing number of healthcare professionals, young and highly educated patients, and the general public using health apps (31–34). Evidence suggests that the use of mHealth apps can play an important role in patient education, disease self-management, remote monitoring of patients, and improvement of quality of life (35–37). For example, LianHong Wang et al. demonstrated that the Transtheoretical Model (TTM)-based mHealth apps can reduce body mass index and other physiological indicators in patients with polycystic syndrome, and improve the exercise and dietary adherence of polycystic syndrome patients in the long term (31–34). syndrome patients’ exercise and diet adherence (38). Basch et al. found that a convenient electronic system for cancer patients to query symptom outcomes could lead to early detection of symptoms and prompt clinicians to intervene (39). Therefore, we believe that mHealth intervention models can be well suited to the postoperative self-management environment for patients with differentiated thyroid cancer, which effectively reduces the stress on healthcare providers and improves patient recovery (40), and reduces patients’ fear of recurrence (41). In public health research, many interventions have helped to improve patients’ self-management level, but there is no study that uses the health theory behavior model as a guide to intervene in the self-health management level of differentiated thyroid cancer patients after surgery. In this study, we will use the WeChat platform as an intervention vehicle for mobile health interventions for DTC patients, with the aim of improving the health behaviors of DTC patients and the corresponding clinical outcomes.

**Figure 1:**
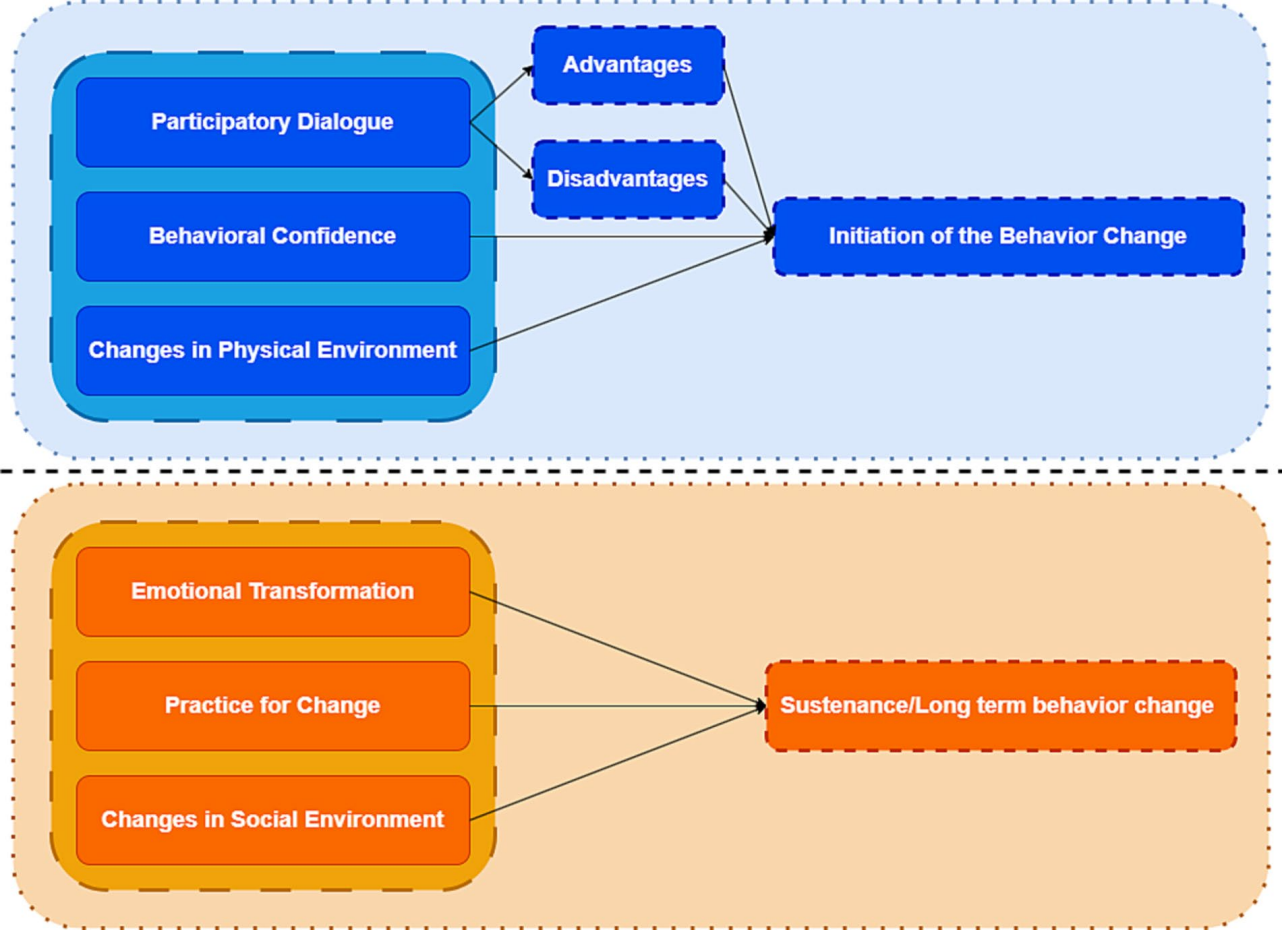
Schematic diagram of a multi-theoretical model of health behavior change.

Health behaviors are positive actions taken by individuals to prevent disease and maintain their own health, which include changing health-hazardous lifestyles, reducing or stopping health-risk behaviors (e.g., smoking, alcohol abuse, poor diet, and unprotected sexual behavior, etc.), adopting positive health behaviors (e.g., regular exercise, regular medical checkups, etc.), and complying with medical advice (42, 43). Theoretical models of health behaviors are important for disease prevention and detection, treatment and rehabilitation, and the promotion and maintenance of physical and mental health, but no intervention studies related to DTC have used theoretical models of health as a guide. Therefore, we aimed to evaluate a mHealth intervention study based on the Multi-theory model of health behavior change (MTM) (44–46) in anticipation of improving self-management in postoperative patients with differentiated thyroid cancer. The MTM divides behavioral change into two components: (1) initiation of behavior change (2) maintenance of behavior change. The initiation of behavior change is influenced by three factors: Participatory Dialog (PD), Behavioral Confidence (BC), and Changes in Physical Environment (CPE). The maintenance of behavioral change is influenced by three factors: Emotional Transformation (ET), Practice for Change (PC) and Changes in Social Environment (CSE) (Figure 1). Its role as a fourth-generation theoretical model used for health education (47–49) has been demonstrated to play an important role in health behavior change, such as smoking cessation (50), promotion of human papilloma virus (HPV) vaccination behaviors (51), and promotion of physical activity practice (47, 52). However, the application of MTM in the field of differentiated thyroid cancer is still in the exploratory stage, with fewer theoretical studies and applications.

In summary, given the great potential of health intervention models guided by the health behavior theory model for prognostic improvement in health care for a wide range of diseases, this study aims to MTM model to construct a health education intervention model for patients with DTC, and to provide a clinical rationale for mobile health intervention to assist in treatment plans related to prognostic rehabilitation of oncology patients by means of mobile patient-doctor interactive health education as a means of intervention.

Specific objectives are

1. To evaluate the impact of the Multi-theory model of health behavior change (MTM) interventions on clinical outcomes in patients with DTC.
2. To determine the effectiveness of the MTM-based intervention program in improving self-management skills of DTC patients.

## 2 Methods

### 2.1 Study design

**Figure 2:**
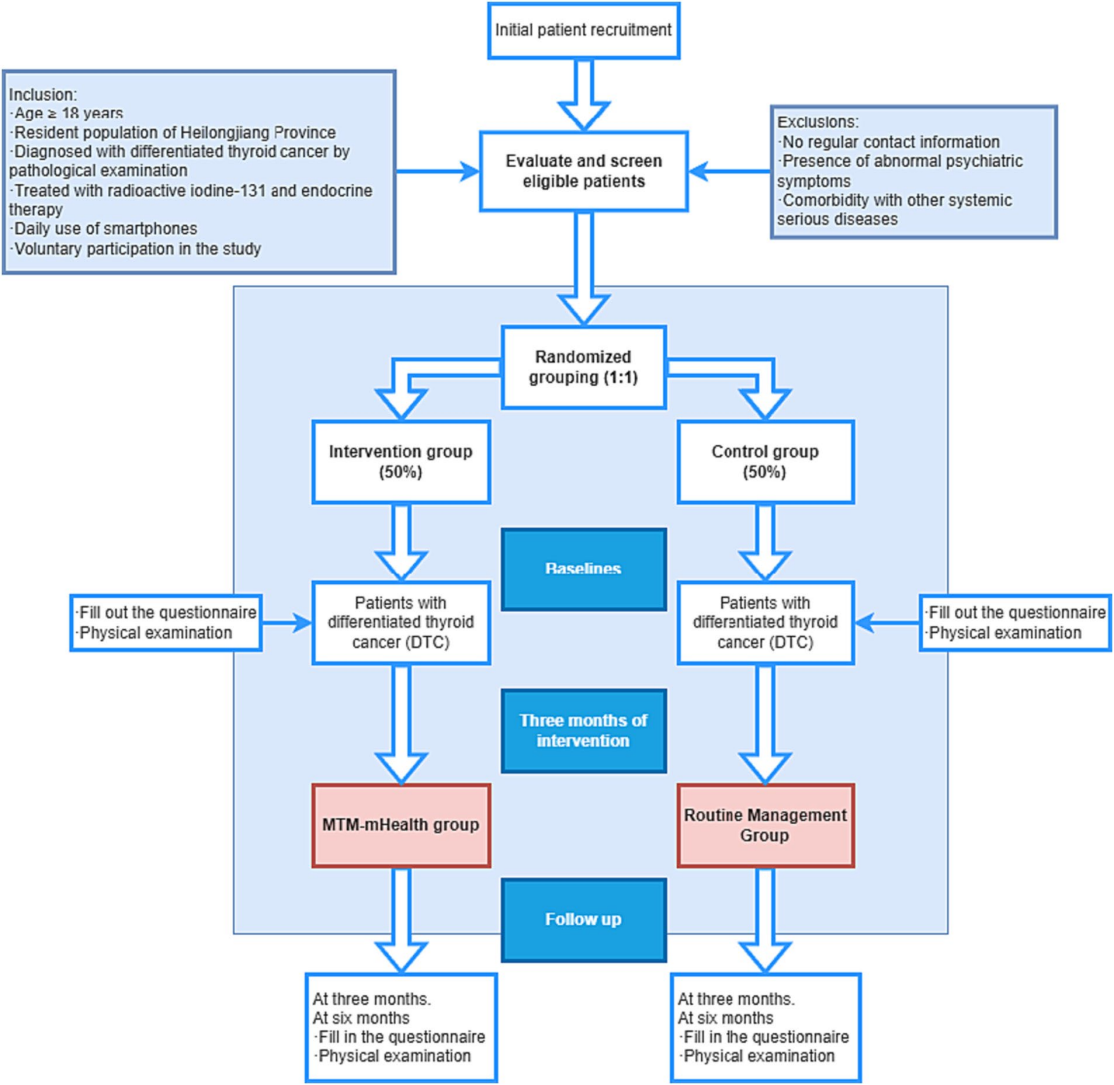
Flow chart of patient recruitment and study implementation.

The study will be conducted from March 2023 to March 2024 using a quantitative methodology study with a measurement protocol that utilizes clinical reagent tests as well as internationally accepted scales to effectively ensure the objectivity of the trial. The quantitative investigation will be conducted as a randomized controlled trial in a hospital-based patient population compared to a routine education control group to compare the effectiveness of the MTM-based intervention program in patients’ TSH control and self-management behaviors (e.g., medication use, diet). The intervention group will receive the MTM-mHealth model intervention and the control group will receive usual care. The intervention will last for 3 months. Questionnaires and physical examinations will be conducted at baseline and at 3 and 6 months of follow-up to check for changes in self-management behaviors and TSH control. The flow chart of the study is shown in Figure 2 and Table 1.

**Table 1:** Timeline for study enrollment, intervention, and evaluation.

Study Period	Recruitment	Allocation	Intervention		Follow-up
TIMEPOINT	-T1	0	0 M	3 M	6 M
ENROLMENT: eligbility screen	√				
Informed consent	√				
Allocation		√			
INTERVENTIONS: MTM-mHealth					
Control					
ASSESSMENTS: sociodemographic variables			√		
Anthropometric variables			√	√	√
The MTM theory scale			√	√	√
The frequency of iodine-containing diets			√	√	√
Medication adherence			√	√	√
Social cognition and psychology			√	√	√

#### 2.1.1 Study setting and randomization

This research project is a single-blind, single-center randomized controlled trial to be conducted at the Fourth Hospital of Harbin Medical University in Harbin, Heilongjiang Province, China. The study population is patients with differentiated thyroid cancer. Convenience sampling of patients with differentiated thyroid cancer was performed. Given that the proposed patient population is socially dispersed cancer patients, the patients coming to the hospital are far from meeting the trial needs of the minimum study sample size at one time, so the patients will be recruited in the form of consecutive enrollment, and the researcher will recruit the patients who have high adherence to the treatment when they come to the hospital on the same day. A random integer will be generated by the EXCEL function RANDBETWEEN for each patient registered on that day.

Given that the total number of patients registered at the hospital on the daily visit may be odd or even, in order to ensure the complete realization of randomized allocation, if the total number of registered patients on the day is even, we will use the random integer of two patients as a combination of numbers, and the groups will be compared, and divided into different groups according to the size of the corresponding random number (large, intervention group, small, control group); if the total number of registered patients on the day is odd, after removing the even pairs of patients, the remaining individual patients are judged by the parity attribute of their random numbers (odd, intervention group, even, control group), thus achieving randomized allocation.

### 2.2 Study participants

Participants in this study are consecutive patients admitted to the Fourth Hospital of Harbin Medical University in Harbin, Heilongjiang Province, China from March 2023 to March 2024 with differentiated thyroid cancer. We have begun recruiting study participants from March 2023, and each participant will begin the formal trial intervention after enrollment in the trial through face-to-face interactions at the hospital as well as signing the study informed consent form. Therefore, all patients are not recruited or starting the intervention at the same time, but will all complete the 3-month intervention cycle.

Inclusion criteria:

1. Patients are ≥18 years old.
2. The patients are permanent residents of Heilongjiang Province (annual out-of-home time less than 1 month).
3. The patients all met the relevant diagnostic criteria for thyroid cancer in the Guidelines for Diagnosis and Treatment of Thyroid Nodules and Differentiated Thyroid Cancer, and were diagnosed with differentiated thyroid cancer by pathological examination.
4. Radiation iodine-131 therapy and endocrine therapy were given after radical surgery for thyroid cancer.
5. The patient uses a smartphone on a daily basis and is familiar with the general function of WeChat.
6. The patient voluntarily participates in the study and signs an informed consent form.

Exclusion criteria:

1. The patient has no fixed contact information, no family member is responsible for contacting the patient, and it is not convenient to contact the patient by phone or WeChat.
2. The patient is taking any psychotropic drugs.
3. The patient has mental symptoms such as delirium, slurred speech, uncooperative, etc.
4. The patient has a combination of other serious systemic diseases, serious physical impairment and participation in other clinical trials.
5. Pregnant or lactating women.
6. Other reasons are not suitable to participate in the trial.

### 2.3 Sample size

This study is a randomized controlled trial, the intervention group is the mHealth intervention group and the control group is the usual care group, the achievement rate of TSH in the study population is the main outcome indicator observed, and the patients’ TSH level control ﹤0.1 mU/L will be completed to achieve the standard. Based on the review of the literature, it is expected that the achievement rate of the mHealth intervention group is 90%, and the achievement rate of the control group is 60%. Setting bilateral *α* = 0.05, the degree of certainty was 90%. Using the PASS15 software, we obtained a sample size of N1 = 39 cases for the treatment group and N2 = 39 cases for the control group, and taking into account the loss of visits and refusal of visits by 20%, the final minimum number of subjects needed for the intervention group and the control group was 49 cases each, for a total of at least 98 subjects to be included in the study.

### 2.4 Treatment programs

Patients in both groups will receive administration of iodine 131 therapy combined with thyroid hormone replacement therapy (53, 54). Prior to receiving iodine 131 therapy, patients will be required to undergo a thyroid uptake scan to determine if there is any residual or recurrent thyroid cancer (55). Depending on individual circumstances, patients are administered iodine 131 between 50 and 200 meters Curie (mCi) in a separate isolation room within the hospital. After administration, patients are hospitalized in an isolation room for 3 days to minimize radiation hazards, and are kept away from pregnant women and small children, avoiding close contact with other people, etc., to ensure safety. After iodine 131 treatment, start to take oral levothyroxine sodium tablets on the second natural day, generally the initial dose is 50 μg per day, the maximum amount does not exceed 100 μg, and the maintenance dose is 50–200 μg per day. Four weeks later, review the thyroid function index, and according to the results of the test, the dose will be maintained or adjusted. Therapeutic target: TSH 0.3 ~ 0.5 mU/L, free thyroxine (FT4) 10.3 ~ 25.8 pmol/L, free triiodothyronine (FT3) 2.16 ~ 6.78 pmol/L.

### 2.5 Interventions

The research team formed a multidisciplinary team consisting of a thyroid cancer pharmacist, a nursing specialist, a thyroid cancer physician, and a public health specialist to discuss and develop the intervention program. The control group received routine medical and nursing care, and the intervention group received the MTM-based mHealth intervention in addition to routine medical care.

#### 2.5.1 Intervention group: online MTM-based health education

The intervention group receives MTM-mHealth, and the realization of health education will rely on the smart terminal WeChat platform. Combined with the chat communication function of WeChat, timely communication between physicians and patients will be realized, and patients’ concerns will be answered by physicians or professional nurses in a timely manner. At the same time, through the WeChat platform, physicians or professional nurses can realize timely interventions for patients, including daily reminders of medication taking, popularization of thyroid cancer-related science, and interpretation of cutting-edge research on thyroid cancer.

The intervention group had WeChat group health education, online health education lectures, received routine medical care such as health education program and medication supervision on the day of hospitalization, then received treatment in the outpatient clinic, and received intervention in mobile health education after discharge for 3 months, and the content of the intervention was designed based on the theory of MTM, and the flowchart is shown in Figure 2.

##### 2.5.1.1 Participatory dialog (when strengths outweigh weaknesses)

Purpose: to motivate patients to start behavioral change, so that patients produce initial action self-efficacy.

Measures: Give each patient a copy of the booklet “Health Education for Thyroid Cancer Patients.” While distributing the education booklet, the physician explains and emphasizes the common problems of the patients, encourages the patients to face the disease positively, and guides the patients to consciously improve their own health behaviors. During the first visit, patients and physicians ask each other questions as much as possible, encouraging patients to actively participate in the initial patient-physician communication. At the same time, the physician added the patient’s WeChat contact information, in order to further provide timely health education to the patient.

After the patient is discharged from the hospital, the physician and the patient regularly communicate with each other through the online doctor-patient salon at 9:00 a.m. on Saturdays, the content of the communication includes disease cognition, prognosis and development, etc., and the link includes the physician’s explanation, the physician-patient Q&A, and the patient–patient communication, etc., and the physician needs to guide the patient to explore the advantages and disadvantages of improving the self-health behaviors on the recovery of the disease, and the physician needs to emphasize that the advantages of the self-health behaviors outweigh the disadvantages so that the patient The physician should emphasize that the advantages of self-health behaviors outweigh the disadvantages, so that the patient can actively participate in the joint decision-making process.

##### 2.5.1.2 Behavioral confidence (belief in one’s ability to achieve desired behaviors)

Purpose: to enhance patients’ confidence in changing health behaviors in the future.

Measures: Physicians and relevant nursing staff set up a doctor-patient exchange group on WeChat, grouping patients according to the initial treatment ward and electing a group leader, who organizes regular exchanges between patients, with the group leader deciding the time of exchanges himself/herself. Physicians and related nursing staff encouraged and instructed all patients to fill out a “health behavior diary” to record the daily maintenance of health behaviors and problems and experiences encountered during the process, and to share and exchange them in the WeChat group every day. At the same time, patients were encouraged to communicate with their physicians in a timely manner, and the physicians evaluated the progress of the patients’ health behaviors in a timely manner. With half a month as a cycle, physicians or relevant nursing staff after evaluation and comparison, the merit of inviting patients who maintain good health behavior to present themselves, in the form of WeChat group to open a patients’ club, the patients through the group learning and communication, sharing daily experience, discussion, interaction and mutual learning.

##### 2.5.1.3 Changes In The physical environment (environments that enable behavior)

Purpose: Provide patients with educational resources in an online format to make resources that support patient health behavior change more accessible.

Measures: Physicians as well as relevant nursing staff need to emphasize with patients and their families at the time of patient discharge and during online communication that common salt should be used to replace iodized salt as much as possible in daily life, educate patients to control the intake of iodine-containing foods, avoid high iodine diets, such as seaweeds like kelp, seaweed and sea fish, and refrain from drinking stimulating beverages such as coffee, strong tea, and cola, and that they should be given diets that are high in calories and contain sugar, proteins, and B-vitamins.

Based on the “Health Education for Thyroid Cancer Patients” manual, physicians and related nursing staff will independently design a delivery program for educational materials, which will be based on an independently constructed multimedia database, and the health education materials in the multimedia database will be evaluated and screened by several related experts before inclusion. The push program will take every 3 days as a push cycle, and push three or more pieces of thyroid cancer related educational materials to patients, which will include video, graphics, voice explanations, etc. The content will include popularized articles about the disease process, interspersed with relevant information about the past cases, the danger of thyroid cancer, and the adverse outcomes of the later stages of the development of thyroid cancer, etc. Physicians can cooperate with the related materials to explain the health behavior. Physicians can explain the necessity and effect of healthy behaviors to improve the adverse outcomes, and guide patients to establish risk awareness. Nursing staff will encourage patients to punch cards in the WeChat group every day, so that they can effectively read the relevant popular science articles.

##### 2.5.1.4 Emotional transformation (intention to overcome self-doubt)

Purpose: To guide the patient’s feelings and emotions to focus on changes in health behaviors, and to guide thoughts to maintain such changes.

Measures: Physicians or relevant nursing staff will remind patients of the health behaviors they need to pay attention to in their lives on the WeChat platform at 9:00 p.m. every day. Relevant texts will be edited in advance and archived in the missionary material push plan, and reminders will be given according to the relevant plan. Physicians encourage and help patients to develop long-term health behavior improvement plans. For those with low adherence: focus on behavioral reinforcement and supervision, repeatedly emphasizing to patients the dangers of thyroid cancer and the benefits of maintaining healthy behaviors, the benefits of taking medication on time, etc.; for those with intermediate levels: focus on praise and encouragement to help them maintain confidence; for those with high levels: focus on monitoring. Physicians need to take the initiative to solicit the recovery effect of all patients, guide patients to take levothyroxine sodium tablets accurately, inform patients of the precautions to be taken after radioactive iodine-131 treatment in a timely manner, and in response to the adverse reactions of the patients after radioactive iodine-131 treatment, patients should be pacified in a timely manner and encouraged to cooperate with the treatment positively.

If patients have negative emotions, resist or discontinue health behaviors during treatment, physicians should promptly analyze the reasons for the discontinuation of their behaviors, take corresponding measures, and guide patients to carry out self-regulation, so that they can quickly resume healthy behaviors despite the discontinuation of their behaviors in a timely manner.

##### 2.5.1.5 Practice change (assessing and adjusting efforts to achieve desired behaviors)

Purpose: To guide the patient in reflective action and make the patient think about the impact of his or her health behavior change.

Measures: The physician regularly organizes a patient exchange salon on Saturday mornings at 9:00 a.m. every week, where patients share their “health behavior diaries” with each other, the daily maintenance of health behaviors and the problems encountered during the process, experiences, etc. For those with low compliance: focusing on the reinforcement of behaviors and urging, the physician needs to discuss the reasons with the patient and help him or her to find a specific solution. For those with low adherence: focus on behavior reinforcement and supervision, the physician needs to discuss the reasons with the patient and help him/her find specific solutions, and emphasize to the patient the disadvantages of abandoning the maintenance of healthy behaviors; for those with medium level of adherence: focus on praise and encouragement, and help him/her maintain confidence; for those with high level of adherence: focus on monitoring.

##### 2.5.1.6 Social environment change (using positive relationships to achieve desired behaviors)

Purpose: construct timely incentives for patients to change health behaviors.

Measures: Physicians and patients communicate with each other in a timely manner on the WeChat platform to understand their psychological state, and combine psychological massage method, verbal appeasement method, and distraction method to motivate patients to make changes and alleviate their poor psychological state through good cases. For those who maintain good health behaviors, timely encouragement is provided in the WeChat group; for those who maintain poor health behaviors, physicians need to communicate with them on a one-to-one basis in a timely manner, take appropriate measures, and guide the patients to self-regulation, so that they can quickly restore healthy behaviors even in the event of behavioral interruptions in a timely manner.

#### 2.5.2 Control group: routine care

Routine discharge education will be given to the control group at the time of discharge, and patients will be given a booklet entitled “Health Education for Patients with Thyroid Cancer,” which includes basic knowledge of thyroid cancer and lifestyle guidance, such as rational diet, medication guidance and adverse reactions. Regular telephone follow-up will be given to the patients, the first telephone follow-up will be given at the end of the first month after discharge and monthly thereafter.

### 2.6 Collection and screening of health education materials

#### 2.6.1 Sources of health education materials

The educational materials used for health education come from the Internet, including videos and illustrations. The illustrations come from WeChat, Wikipedia, doctor’s Q&A, or domestic and international databases such as CNKI, and the videos come from WeChat, Wikipedia, doctor’s Q&A, etc. The cases come from Baidu documents or literature. The case information was obtained from Baidu documents or literature, and the educational materials used should be scientific and have corresponding references.

#### 2.6.2 Screening of health education materials

After centralizing all the health education materials, they will be sent for external review to the experts in thyroid cancer related fields, who will evaluate the materials according to the thyroid cancer expert evaluation form. After the experts return the evaluation opinions, they will centralize the screening of the quality health education materials, and the selected health education materials will be used as the push database of the health education materials. The push database will contain article titles, related links, corresponding sources, corresponding material types, etc. The materials will be categorized according to their corresponding contents. The materials will be categorized into disease awareness, postoperative care, diet, exercise, and other sections according to their corresponding content, so as to facilitate the formulation of a corresponding delivery plan for patients.

### 2.7 Outcomes

Primary Outcome: Change in TSH from Baseline and at 3 and 6 Months Follow-Up.

Secondary outcomes:

1. changes in self-management behaviors: changes measured by the MTM Theory Scale, changes in frequency of iodine-containing diets, medication adherence.
2. Social cognitive and psychological: quality of life, satisfaction, depression, anxiety and fear of cancer recurrence.
3. Metabolic control: thyroxine (T4), triiodothyronine (T3), FT3, FT4.

The corresponding study questionnaires completed by all patients at baseline as well as at follow-up were piggybacked on a web-based platform (Questionnaire Star). Clinical pharmacists distributed the questionnaires via Questionnaire Star, and patients were instructed to fill them out by pharmacists either face-to-face (during hospitalization) or by telephone (after discharge). Patient demographic information was collected through the case system and self-designed questionnaires.

### 2.8 Variables measurement

Socio-demographic variables will be collected: gender, age, education, usual residence, *per capita* monthly income of the family, mode of bearing medical expenses, and clinical information such as duration of DTC, detailed medical history.

Physiological Indicator Variables: TSH will be detected using radioimmunoassay (RIA), the medical staff will clean the patient’s blood collection site using a sterilized cotton ball, and then use a needle to take an appropriate amount of fasting venous blood 5 mL, centrifuged at 3000 r/min for 5 min, serum will be separated, and a certain amount of TSH antibody labeled with the radioisotope iodine-125 will be added to the processed serum to bind to the TSH to be detected. TSH. Using biochemical techniques, the antibody-bound TSH and unbound free TSH were separated into solid phase and free phase. The radioisotopes in the solid phase are measured to obtain a corresponding count value, from which the concentration of TSH in the sample can be determined. Based on the stratification of risk factors for recurrence of differentiated thyroid cancer, patients can be categorized into low-risk, intermediate-risk, and high-risk strata, and postoperative serum TSH target values are set accordingly (56). In general, the division is based on factors such as age, gender, tumor stage, presence of lymph node metastasis, and type of thyroid cancer variant. All patients included in this trial were high-risk patients treated with iodine 131, and according to treatment guidelines, for high-risk patients, postoperative serum TSH should be suppressed to very low levels (<0.1 mIU/L) or completely suppress thyroid function to minimize the risk of recurrence. Therefore, the TSH compliance rate was defined as postoperative serum TSH < 0.1 mU/L, and this criterion was used to calculate the TSH compliance rate.

T4, T3, FT3, and FT4 will be measured by enzyme-linked immunosorbent assay (ELISA). The medical staff will clean the patient’s blood collection site using a sterilized cotton ball, and then use a needle to take a tube of appropriate amount of fasting venous blood of 5 mL, respectively, to prepare the required reagents, including enzyme-labeled antibody, substrate, and washing buffer. At the same time, prepare the sample to be tested. Fix the specific antigen or antibody on a microtiter plate, usually using a multiwell plate, such as a 96-well plate. Add enough antigen or antibody solution to each well and incubate at a constant temperature so that it adsorbs to the well walls. The samples to be tested are pretreated as necessary, e.g., dilution, protein cleavage. The pre-treated samples or standards are added to each well. A series of standard curve samples of varying concentrations are usually set up and used to generate a standard curve to calculate the concentration of the target in the sample to be tested. Enzyme-labeled antibodies conjugated to the target are then added and incubated. The samples and unbound material in the wells are washed away to minimize non-specific binding. An appropriate substrate is added to react with the enzyme-labeled antibody. The choice of substrate depends on the enzyme marker used, commonly horseradish peroxidase (HRP) used in ELISA. After an appropriate period, the reaction is terminated by the addition of a reaction stopping solution and prevented from producing further color changes. The absorbance values of each well are read using an enzyme meter or photometer. Based on the standard curve, the concentration of the target in the sample to be tested is calculated.

Sociodemographic variables will be collected from patients at baseline. Clinical reagent testing and questionnaires will be completed at baseline as well as at 3 and 6 months of follow-up, with data collection times shown in Table 2.

**Table 2:** Data collection components and collection timeline.

Type of research	Data collection component		Timepoint		
			0 M	3 M	6 M
Quantitative research	Sociodemographic variables	Sex, age, social insurance status, place of residence, average monthly household income, educational level, area of domicile, clinical information	√		
	Anthropometric variables	Thyroid stimulating hormone (TSH), thyroxine (T4), triiodothyronine (T3), Free T3, Free T4	√	√	√
	The MTM theory scale	Questionnaire: Changes measured by the MTM Theory Scale	√	√	√
	Tthe frequency of iodine-containing diets	Questionnaire: Changes in the frequency of iodine-containing diets	√	√	√
	Medication adherence	Questionnaire: Medication Refill or Medication Adherence Scale (ARMS-7)	√	√	√
	Quality of life	Questionnaire: EuroQol-5 Dimensions 5 Levels(EQ-5D-5 L7)	√	√	√
	Satisfaction	Questionnaire: Customer Satisfaction Questionnaire (CSQ-3)	√	√	√
	Depression and anxiety	Questionnaire: Simplified version of the Anxiety Depression Scale (PHQ-4)	√	√	√
	Fear of cancer recurrence	Questionnaire: Fear of Cancer Recurrence Scale (FCR-4)	√	√	√

### 2.9 Questionnaires

Changes in self-management behaviors will be assessed by a self-designed MTM scale (57) consisting of 6 dimensions including PD, BC, CPE, ET, PC, and, CSE using a 5-point Likert scale design, the scale will be developed and final validation will be completed by the researcher, and 20 randomly selected participants who did not take part in the final trial will complete a pre-test from the recruited participants to determine the appearance validity and the content validity through pre-testing and expert testing to refine the model scale.

Changes in the frequency of iodine-containing diets will be assessed by patients through a self-administered the Iodine-containing Food Frequency Questionnaire (I-FFQ) questionnaire after adaptive adaptation and refinement based on the Food Frequency Questionnaire (FFQ) (58, 59). Preparation will be done in consultation with experts and based on food guidelines, several foods likely to be consumed on a daily basis with an iodine value greater than 400 mg/kg (60, 61), including iodized salt, will be selected as the target for measurement, and the time scale will be measured as the basis for frequency (62).

Medication adherence will be measured using the Renewal or Medication Adherence Scale (ARMS-7), which has seven entries and two dimensions: medication adherence (entries 1–4) and refills (entries 5–7), which is a 4-point Likert scale ranging from 1 (none) to 4 (all), notably one entry in the scale (the seventh) is reverse scored (1 = all to 4 = none). It measures self-reported medication adherence with respect to both taking medication as prescribed and taking medication as scheduled. The total score was derived by summing the responses to all items. The total score ranges between 7 and 28, with higher scores being associated with lower medication adherence. The scale has high validity and reliability in measuring medication adherence, with total scale item correlation coefficients ranging from 0.35 ~ 0.58 and a Cronbach’s alpha of 0.75 (63).

Quality of life will be assessed using the EuroQol-5 Dimensions 5 Levels (EQ-5D-5L), which is an improved version of the EQ-5D that introduces five tiers (64, 65) on top of the original five dimensions, (eg. no difficulty, minor difficulty, moderate difficulty, major difficulty, total difficulty) thus providing a more refined health status Assessment. Its health utility values ranged from 0.62 to 0.90 in seven studies reporting cancer patients with high validity and reliability (66–72). Compared to the traditional EQ-5D (EQ-5D-3L), the dimensional and hierarchical structure of the EQ-5D-5L remains unchanged, but the number of ratings for each dimension has been increased to five. The five dimensions of the EQ-5D-5L include Mobility (Mobility), Self-care (Self-care), Usual activities (Usual activities), Pain/Discomfort, and Anxiety/Depression. For each dimension, the individual selects the appropriate level according to his or her situation, thus constituting a five-dimensional description. In addition to the five-dimensional descriptors, the EQ-5D includes a Visual Analog Scale, which allows individuals to give a subjective rating of their current state of well-being based on their feelings on a scale of 0 to 100. This score provides a continuous variable to measure an individual’s overall quality of life.

Satisfaction will be adapted using the Customer Satisfaction Questionnaire (CSQ-3), which is a commonly used customer satisfaction questionnaire for assessing customer satisfaction with a product, service, or experience, with a Cronbach’s alpha of 0.84, and good reliability and validity (73, 74). The CSQ-3 is a revised version of the CSQ which was developed by Roger, D et al. in 1993 and has been widely used in the fields of market research, customer relationship management, and business decision making (75, 76). This question is designed to assess the customer’s level of satisfaction with the overall product, service, or experience. Response options are usually on a five-point scale from very dissatisfied to very satisfied. The CSQ-3 is designed to be simple and straightforward and is suitable for quickly gathering information on customer satisfaction.

Depression and anxiety will be assessed using a simplified version of the Anxiety Depression Scale (PHQ-4), which is a short, self-report scale used to assess an individual’s symptoms of depression and anxiety over the past 2 weeks, and consists of two dimensions of depression as well as anxiety, with a total of four entries, the first two of which relate to depressed mood and the last two to anxiety (77). Each entry was rated on a 4-point scale, with “not at all, ““a few days,” “more than half the days,” and “almost every day,” corresponding to 0, 0, and 0, respectively. Each entry is rated on a 4-point scale, with “not at all,” “a few days,” “more than half the days,” and “almost every day” corresponding to a score of 0, 1, 2, and 3, respectively, and the total score ranges from 0 to 12. The Cronbach’s alpha of the PHQ-4 was 0.833, which is of high validity and reliability, in a study conducted in China (78). The PHQ-4 scale is very concise, and it is easy to use and comprehend. It can help healthcare professionals to quickly screen individuals with symptoms of anxiety and depression and provide an initial assessment.

Fear of cancer recurrence will be assessed using the Fear of Cancer Recurrence Scale (FCR-4) (79). The FCR-4 scale is a simplified version based on the “FCR-7” scale, developed by Simard and Savard in 2018, with a measured Cronbach’s alpha of 0.86 (80). The FCR-4 consists of four questions to assess the level of fear of cancer recurrence among cancer patients. Recurrence and is usually rated on a five-point Likert scale from completely disagree to completely agree. The use of the FCR-4 scale can help healthcare professionals to understand the level of fear cancer patients have about cancer recurrence and provide them with appropriate support and mental health resources. This information can be used in the development of individualized treatment plans to help patients cope with their fears and improve their quality of life.

## 3 Statistical analysis

Statistical analysis was performed using SPSS Statistics 26.0 software. Count data were expressed as a number of cases and rate (%). The distribution of continuous data will be tested for normality, and information conforming to normal distribution will be expressed as mean ± standard deviation (M ± SD), and non-normal information will be expressed as median (IQR). The Mann–Whitney U test will be used to compare the change in scale scores between baseline and endpoint between the two groups, and the Wilcoxon test will be used to compare the difference in scale scores between baseline and endpoint between the two groups. The chi-square test will be used to compare the demographic characteristics of the two groups. A two-sided test will be used and will be considered statistically significant if *p* < 0.05.

Assuming a reasonable amount of missing data, the data summary will indicate that data are missing at random. In this case, all analyses will be performed with the baseline variable as the auxiliary variable, the missing data will be supplemented by multiple interpolation procedures, linear regression and logistic regression will be selected for the analyses, and the final combined analyses will be performed to complete the process of interpolation and analysis.

### 3.1 Study management

#### 3.1.1 Data collection

Prior to the commencement of the study, the investigator will be fully trained and assessed. The investigator will need to ensure that all study results, including clinical and laboratory data, will be recorded in a pre-made subject registration form so that study subjects can be enrolled and coded to minimize the incidence of missed visits during follow-up. To maintain consistency between the intervention and control groups, survey times and questionnaires will remain essentially the same. The investigator will need to ensure that all sections of the registration form are entered correctly and that each completed patient’s data must be dated and signed to ensure that the trial can be validated retrospectively based on the data once completed. During subsequent data analysis, a double-checking procedure will be implemented to improve the quality of data entry, and experts will be consulted to select appropriate statistical methods.

#### 3.1.2 Storage and archiving of data

Investigators are required to archive all trial data (list of subject identification codes, source data, and investigator files) and related correspondence in study-specific database files. All source data for the database and all related documentation for the study will be archived in accordance with laws and regulations after the trial is finalized.

#### 3.1.3 Ethics and regulations

The procedures set out in this trial protocol relating to the conduct, evaluation and documentation of this trial are designed to ensure that all participants in the trial adhere to the ethical principles described in GCP and the current revision of the Declaration of Helsinki. The trial will be conducted in accordance with the requirements of local laws and regulations, and approval for this trial protocol was obtained from the Ethics Committee of the Fourth Hospital of Harbin Medical University (2022-WZYSLLSC-20).

#### 3.1.4 Ethics committee

For quality assurance, this trial will comply with ethical and legal requirements and the trial will have to inform the Ethics Committee of all subsequent protocol revisions that require formal approval in accordance with local legal requirements. The Ethics Committee will have to be informed in advance of the trial procedures, if not specified more in the documentation. The Ethics Committee will have to be informed of the end of the trial at the end of this trial.

#### 3.1.5 Informed consent

Prior to undergoing the trial, patients will have to voluntarily agree to participate after an explanation of the nature, scope and possible consequences of this trial. Informed consent must be obtained from each individual patient or their legally authorized representative prior to the start of the trial. Patients who are unable to sign but are able to understand the significance of participation in the study may give verbally witnessed informed consent. These patients must clearly demonstrate that they are willing to participate voluntarily and must be able to understand an explanation of the contents of the informed consent form.

#### 3.1.6 Confidentiality

Authorized consent to use their trial data must be obtained from the patient prior to enrollment. To protect patient privacy, the patient’s age will be recorded on the registration form without recording the patient’s year of birth, and the name will be replaced by a cell phone number. During the trial, trial results stored on computers will be stored in accordance with local data protection laws and will be handled in strict confidentiality. The investigator will implement organizational procedures that set access rights to the files to prevent data from being sent to unauthorized persons. Appropriate provisions of local data regulations will be fully complied with. Authorized personnel may inspect subject-related data collected during the trial to ensure that the data are legally protected.

#### 3.1.7 Investigator responsibilities

Prior to the start of the trial, the trial protocol, informed consent document and any other appropriate documentation will be presented to the independent study team members. The study leader and principal investigator will regularly supervise the study implementers to ensure that all study procedures are being carried out correctly and that the data collected are accurate. Each investigator should ensure that all personnel assisting with the trial are fully informed about the protocol, any modifications to the protocol, the trial treatment, and their duties and functions related to the trial.

#### 3.1.8 Approval of trial protocol and amendments

If the investigator fails to comply with the pre-established survey and follow-up criteria, the study leader will retain the authority to terminate the trial, revise the randomization protocol, and re-collect the data after the revisions have been completed. In the event of fundamental or widespread design errors or inaccuracies in the study questionnaire during the course of the investigation, the investigator will discontinue the trial and revise and redesign the questionnaire. After the necessary revisions have been completed, the revised questionnaire will be redistributed to patients.

#### 3.1.9 Data monitoring

The Data Monitoring Committee (DMC) will consist of at least two members from the Medical Ethics Committee of the Fourth Affiliated Hospital of Harbin Medical University, who must not have a conflict of interest with the investigators of the project. The DMC will monitor and review the study on a regular basis and report the results to the Ethics Committee. This process will be independent of the principal investigator. The DMC will have the right to suspend or terminate the study if they find any deviation from the approved study protocol or any unauthorized changes in the study procedures during the course of the study.

#### 3.1.10 Safety

All adverse events (SAEs) or adverse events (AEs) reported by the patient or detected by the investigator will be collected during the trial and must be recorded on the appropriate page of the registration form. All SAEs and AEs that occur after the patient has signed the informed consent document will be recorded on the page of the registration form. All patients with SAEs and AEs, whether or not considered relevant to the trial, must be monitored to determine outcomes. This trial will have to be reported to the primary study leader within 24 h of the SAE and AE occurring. The initial report must be as complete as possible, including details of the (serious) SAE and AE in the current trial and an assessment of the causal relationship between the event and the trial and the occurrence of the event. The Principal Study Leader will notify the Trial Supervisor of serious SAEs on request and also has a responsibility to report any serious SAEs or AEs to the Ethics Committee in a timely manner.

## 4 Discussion

The great leap forward in the information age has broken the information gap between “medical” and “personal” for centuries. Obtaining personalized medical knowledge and information through the mobile Internet is an essential skill in this era, and therefore mHealth is rapidly developing. mHealth can be thought of as a healthcare that uses mobile communication devices as a carrier, and supports the long-term sustainability of healthcare by improving effective communication between doctors and patients and the patient’s understanding of the disease based on online communication channels, such as video, telephone, and discussion forums. Both at present and in the foreseeable future, mHealth has shown great potential for development in all kinds of medical scenarios, whether it is in the prognosis and management of chronic diseases, or the construction of rapid treatment solutions for sudden illnesses, which can efficiently realize the deployment of medical resources. In addition, mHealth is more affordable than traditional doctor-patient scenarios, with time and geographic costs close to zero for patients, as evidenced by studies of primary care self-management behaviors related to chronic diseases. However, more authoritative health theories have not been widely applied as theoretical guides for health interventions in common mHealth research, especially in the cancer patient population, which is in greater need of postoperative health care. This study will explore the application of MTM theoretical modeling as a guide for health intervention research in the DTC patient population, with the aim of evaluating MTM-based intervention programs for clinical outcome improvement in DTC patients, as well as determining the effectiveness of MTM-based intervention programs in improving self-management skills in DTC patients. This study is conducive to filling the research gap of postoperative health management of Chinese DTC patients after being guided by health-based theories.

For patients with DTC, especially those who are at high risk of recurrence as determined by the postoperative risk factor stratification, it is especially crucial for them to actively cooperate with the doctor’s prescription for continuous TSH-suppressing endocrine therapy and self-health management after postoperative iodine 131 treatment. However, given that the mortality rate of differentiated thyroid cancer is very low compared with other types of cancer, patients may become lax in the prognosis process, especially the insufficient control of iodine-containing foods in their daily diets, or the erroneous consumption of other foods or beverages that may have an impact on the TSH-inhibiting drugs, which may have a great negative impact on the therapeutic effect. Therefore, it is important to explore more effective ways to improve patients’ self-management in the context of the current information age. In public health research, most mHealth interventions have been helpful in improving patients’ self-management, but there is no research on self-management of DTC patients in the context of mHealth. After combining mHealth, we selected the fourth-generation MTM model, which is a guiding significance for health intervention research in the international frontiers, to improve our intervention pathway and transform the theory into practice. Meanwhile, the application of MTM model in China is in the preliminary stage of exploration, and this study will fill the gap in this direction and provide new ideas for health management programs related to cancer prognosis and rehabilitation. The results of this study will show whether the application of health theory modeling based on health theory combined with mHealth application for disease prognosis health management model is feasible as well as effective, and provide policy recommendations and technical translation for the development of the application of mobility health management in the field of health management.

## Ethics statement

The study has been approved by the Ethics Committee of the Fourth Affiliated Hospital of Harbin Medical University, Harbin, Heilongjiang Province (2022-WZYSLLSC-20), and the registration of the study protocol with the China Clinical Trial Registry (ChiCTR2200064321) has also been completed. The participants provided their written informed consent to participate in this study.

## Author contributions

YJ: Conceptualization, Investigation, Methodology, Writing – original draft, Writing – review & editing. XS: Conceptualization, Investigation, Project administration, Resources, Writing – original draft, Writing – review & editing. MJ: Funding acquisition, Resources, Writing – review & editing. HM: Supervision, Writing – review & editing. JW: Writing – review & editing. XF: Writing – review & editing. JQ: Writing – review & editing. ZY: Writing – review & editing. XZ: Project administration, Supervision, Writing – review & editing. YW: Project administration, Supervision, Writing – review & editing.

## 5 Glossary

**Table:** 

DTC	Differentiated thyroid cancer
MTM	Multi-theory model of health behavior change
PD	Participatory Dialog
BC	Behavioral Confidence
CPE	Changes in Physical Environment
ET	Emotional Transformation
PC	Practice for Change
CSE	Changes in Social Environment
mHealth	Mobile health
T4	Thyroxine
T3	Triiodothyronine
TTM	Transtheoretical model
DMC	The Data Monitoring Committee
SAEs	All adverse events
AEs	Adverse events
CSQ-3	The Customer Satisfaction Questionnaire
EQ-5D-5L	The EuroQol-5 Dimensions 5 Levels
ARMS-7	The Renewal or Medication Adherence Scale
FFQ	The Food Frequency Questionnaire
I-FFQ	The Iodine-containing Food Frequency Questionnaire
HRP	Horseradish peroxidase
ELISA	Enzyme-linked immunosorbent assay
HPV	Human papilloma virus
mCi	Meters Curie
FT4	Free thyroxine
FT3	Free triiodothyronine
RIA	Radioimmunoassay
PHQ-4	A simplified version of the Anxiety Depression Scale
FCR-4	The Fear of Cancer Recurrence Scale
