# Corrigendum: Impact of a mobile health intervention based on multi-theory model of health behavior change on self-management in patients with differentiated thyroid cancer: protocol for a randomized controlled trial

**DOI:** 10.3389/fpubh.2024.1414576

**Published:** 2024-04-29

**Authors:** Yang Jiang, Xiangju Sun, Maomin Jiang, Hewei Min, Jing Wang, Xinghua Fu, Jiale Qi, Zhenjie Yu, Xiaomei Zhu, Yibo Wu

**Affiliations:** ^1^Jitang College, North China University of Science and Technology, Tangshan, China; ^2^Clinical Pharmacy, The Fourth Affiliated Hospital of Harbin Medical University, Harbin, China; ^3^School of Public Affairs, Xiamen University, Xiamen, China; ^4^School of Public Health, Peking University, Beijing, China; ^5^The Fourth School of Clinical Medicine, Harbin Medical University, Harbin, China; ^6^School of Journalism and Communication, Zhengzhou University, Zhengzhou, China; ^7^School of Nursing, Tianjin Medical University, Tianjin, China; ^8^Department of Pharmacy, Beidahuang Group General Hospital, Harbin, China

**Keywords:** differentiated thyroid cancer, mHealth, MTM, health education, self-management

In the published article, there was an error. The ChiCTR registration number for the Project was displayed as “ChiCTR2200054321”. The correct number is “ChiCTR2200064321”.

A correction has been made to **[Methods]**, [*Study participants***]**, [paragraph 2.2], and this sentence will now move to a separate end Ethics Statement as per Frontiers format This sentence previously stated:

“The study has been approved by the Ethics Committee of the Fourth Affiliated Hospital of Harbin Medical University, Harbin, Heilongjiang Province (2022-WZYSLLSC-20), and the registration of the study protocol with the China Clinical Trial Registry (ChiCTR2200054321) has also been completed.”

The corrected sentence appears below:

“The study has been approved by the Ethics Committee of the Fourth Affiliated Hospital of Harbin Medical University, Harbin, Heilongjiang Province (2022-WZYSLLSC-20), and the registration of the study protocol with the China Clinical Trial Registry (ChiCTR2200064321) has also been completed. The participants provided their written informed consent to participate in this study.”

The authors apologize for this error and state that this does not change the scientific conclusions of the article in any way. The original article has been updated.

