# Supplementary material for: Impact of a mobile health intervention based on multi-theory model of health behavior change on self-management in patients with differentiated thyroid cancer: protocol for a randomized controlled trial
Source: Front Public Health. 2024 Jan 11;12:1327442. doi: 10.3389/fpubh.2024.1327442 (PMC10808536; doi:10.3389/fpubh.2024.1327442)
Supplement: Supplementary file 1 [file Presentation_1.PDF]

哈尔滨医科大学附属第四医院伦理审查批件

伦审号：2022-WZYSLLSC-20

**伦理审查日期：**	2022 年 8 月 29 日
**试验项目名称：**	基于多理论模型的移动性健康干预对甲状腺癌患者自我管理行为的影响干预性随机对照试验
**主要研究者：**	孙向菊
**审查方式：**	报送材料，审查备案。
**审查文件：**	1. 研究方案<br>2. 知情同意书<br>3. 主要研究者声明

审查意见

该项目完整的医学伦理审查材料于 2022 年 8 月 29 日正式递交于我单位医学伦理委员会，经核实材料齐全，涉及医学伦理审查内容填写完整，同意进行存档备案并后续发表相关文章。请遵循 GCP 及《赫尔辛基宣言》的要求开展临床研究。

本伦理委员会的职责、人员组成、操作程序及记录均遵循 ICH-GCP 以及中国的相关法律法规。

注意事项：

所有资料未经委员会审核批准，不得作任何修改；

试验过程中若发生违背试验方案，及时提交违背方案报告；

暂停或提前终止研究，及时提交暂停/终止审查申请。

哈尔滨医科大学附属第四医院医学伦理委员会

（盖章）

伦理委员会地址：黑龙江省哈尔滨市南岗区银行街 31 号　　联系方式：82576558
