# Supplementary material for: Impact of a mobile health intervention based on multi-theory model of health behavior change on self-management in patients with differentiated thyroid cancer: protocol for a randomized controlled trial
Source: Front Public Health. 2024 Jan 11;12:1327442. doi: 10.3389/fpubh.2024.1327442 (PMC10808536; doi:10.3389/fpubh.2024.1327442)
Supplement: Supplementary file 2 [file Data_Sheet_1.DOC]

**知情同意书**

**第一部分 受试者须知**

我们将要开展一项（“基于多理论模型的移动性健康干预对甲状腺癌患者自我管理行为的影响”）研究，您符合该项研究的入组条件，因此，我们想邀请您参加该项研究。本知情同意书将向您介绍该研究的目的、步骤、获益、风险、可能给您带来的不便或不适等，请仔细阅读后慎重做出是否参加该研究的决定。当研究人员向您说明和讨论知情同意书时，您可以随时提问并让他/她向您解释您不明白的地方。您可以与家人、朋友以及您的主治大夫讨论之后再做决定。

若您目前正参加其他临床研究，请务必告知您的研究医生或者研究人员。

本项研究的项目负责人是哈尔滨医科大学附属第四医院孙向菊。

1. **研究背景和研究目的：**

甲状腺癌的发病率不断上升，其中分化型甲状腺癌(DTC)，包括乳头状癌和滤泡状癌，占所有甲状腺癌的绝大多数(>90%)。奥姆斯特德县最近的一项基于人群的研究报告称，与前十年相比，2000年至2012年甲状腺癌发病率翻了一番。甲状腺癌患者的预后范围变化很大，间变性甲状腺癌是最致命的实体肿瘤类型之一。随着医疗技术的进步，甲状腺肿瘤患者对疾病管理的个性化、精准化需求日益增加。但由于人力、能力等原因，部分基层医疗卫生机构及医务人员对于甲状腺肿瘤患者的干预管理效果欠佳。在移动健康蓬勃发展趋势下，如何让移动健康真正助力疾病的预防和控制，是个值得研究的课题。

本试验的目的是为了探索移动性健康干预是否能改善甲状腺癌患者自我管理行为以及相应的临床指标

1. **研究简介：**

该项研究的设计方法为干预性随机对照，主要研究移动性健康干预是否能改善甲状腺癌患者自我管理行为以及相应的临床指标，比较基于移动健康干预模式与传统管理模式在人群中产生的效果差异，从而为改善甲状腺癌患者的自我管理行为、帮助控制病情以及死亡风险等干预措施提供相应依据，探究出一套借助移动智能终端的、可推广、可持续的甲状腺癌患者健康教育干预模式。预期参加的患者人数98人。

您将被随机分配到两个不同组别的其中一个，包括移动健康教育管理组以及常规管理组。整个分配流程是完全随机的。

移动健康教育管理组将接受移动性健康教育，通过微信的聊天通讯功能，将实现医师与您之间的及时沟通，您的疑虑将得到医师或专业护士的及时解答，同时通过微信平台，医师或专业护士可实现对您的及时健康管理，包括服药的日常提醒，对甲状腺癌相关的科普，关于甲状腺癌的前沿研究的解读等。

常规管理组出院时将给予常规出院教育，将向您发放甲状腺癌健康教育手册，其内容包括甲状腺癌基本知识和生活方式指导，如合理饮食、药物指导和不良反应。研究人员将对您定期进行电话随访，出院后第一周结束将进行第一次电话随访，此后每月一次电话随访。期限为90天，随访3次。

所有患者都将接受所有必要的诊断或治疗程序，包括根据当前的《甲状腺结节和分化型甲状腺癌诊治指南》进行康复治疗，以及应有的健康知识教育。

1. **试验入选标准**

·年龄≥18岁

·为黑龙江省哈尔滨市常驻人口（年外出时间小于 1 个月）

·均符合《甲状腺结节和分化型甲状腺癌诊治指南》中关于甲状腺癌的相关诊断标准，经病理学检查确诊为分化型甲状腺癌

·接受甲状腺癌根治性手术后均给予放射性碘治疗治疗

·日常使用智能手机，及熟悉一般微信功能；

·自愿参加研究，签署知情同意书

1. **试验排除标准**

·无固定联系方式、无家属负责联系、不方便电话或微信联系

·服用任何精神类药物

·存在神志、语言表达不清、不合作等精神症状

·合并其他系统严重疾病、严重躯体功能损害及参与其他临床试验

·妊娠或哺乳期妇女

·其它原因不适合参与试验者

1. **受试者的责任**

作为参与研究的受试者，您有以下职责：提供有关自身病史和当前身体状况的真实情况；告诉研究医生自己在本次试验期间所出现的任何问题；不得服用受限制的药物、食物等；告诉研究医生自己在最近是否曾参与其他研究，或目前正参与其他研究等。

1. **参加研究可能的受益**

1.研究对您本人的益处

本研究可能会治愈疾病或减缓疾病的发展，但是我们不能对此作出保证。本研究中的健康教育知识对您了解自身病情有重要帮助，虽然不能直接缓解病情，但是能够帮助您解答各类疾病的相关问题，持续从中获益。

2.研究对社会群体可能的益处

尽管参加本次试验可能不会给您带来直接的益处，但您的参与可能会给未来遭受同样痛苦的您带来益处。

1. **参加研究可能的不良反应、风险以及不适**

本次试验对您没有经济方面的损失，但调查问卷会花费您较少时间，某些问题可能引起您的不适以及存在个人隐私泄露的风险，同时可能存在某些研究者无法预知的风险。本试验无不良反应。本试验将对您填写的信息绝对保密，在资料管理上有严格的审查标准。

1. **相关治疗和补偿**

您如果在此期间出现不适或其他反应，请及时告知医生，医生将根据情况及时采取相应的措施。该试验不会对您的治疗方案造成影响，仅属于健康教育类研究，这对您获得常规治疗不会带来任何不良影响。

1. **可以自愿选择参加研究和中途退出研究**

您的参加与否完全自愿，如果您自愿参加并成功入选，我们希望您能坚持完成本试验；您可不需任何原因随时决定不参与或中途退出此项试验，退出时我们会为您进行健康评估，如有异常将会继续随访至您恢复正常或至稳定阶段。若您退出试验，我们保证您不会遭到歧视或报复，您的任何医疗待遇与权益不会因此而受到影响。

1. **个人信息保密**

如果您决定参加本项试验，您参加试验及在试验中的个人资料均属保密。负责研究医师及其他研究人员将使用您的医疗信息进行研究。这些信息可能包括您的姓名、地址、电话号码、病史及在您研究来访时得到的信息。您的档案将保存在有锁的档案柜中，仅供研究人员查阅。研究中会用编号来标识您的研究信息和试验室检查标本。只有研究者和研究小组成员可查询编号。为确保研究按照规定进行，必要时，研究申办者，政府管理部门或伦理审查委员会的成员按规定可以在研究单位查阅您的个人资料。这项研究结果发表时，将不会披露您个人的任何资料。

1. **怎样获得更多的信息？**

研究期间，如果得到可能影响您继续参加试验的信息，您或其合法代理人将及时得到通报，必要时将向您获取新签署的知情同意书。

您可随时了解与本试验有关的信息资料和研究进展，如果您有与本试验有关的问题，或您在研究过程中发生了任何不适与损伤，或有关于本项研究参加者权益方面的问题您可以通过**13836168134**与孙向菊联系。

1. **相关咨询**

该研究方案经过哈医大四院医学伦理委员会批准，研究过程中有任何违反研究方案的情况，或您有与自身权益相关的任何问题，或您想反映参与本研究过程中的不满和忧虑，您可以向伦理委员会申诉。

哈医大四院医学伦理委员会办公室电话：

哈医大四院医学伦理委员会电子邮箱：

联系人：王玉珏（秘书）

**在您（或您的法定代理人）决定加入本临床试验/研究之前，请认真阅读此知情同意书，研究医生将帮助为您解答有关受试产品及与此项试验/研究相关的问题。如果您自愿参加，在阅读完这些资料后，请您在知情同意书的最后一页签署名字和日期。**

**第二部分 知情同意签名**

1. 我已认真阅读了本研究项目的知情同意书中您须知的内容，研究医生已向我做了详尽说明并解答了我的相关问题，我完全了解参加本次试验/研究的目的、过程及我的权益和风险。 我自愿参加本次试验/研究，并同意按照知情同意书的内容配合研究医生进行治疗和随访，尽力完成本次试验/研究。

受试者签字：

*（印刷体）* （手写体） 日期

或受试者法定代理人签字（必要时）：

*（印刷体）* 与您关系：

（手写体） 日期

2. 我或我的研究人员已向该您充分解释和说明了本试验/研究的目的、操作过程以及您参加该试验/研究可能存在的风险和潜在的获益，并满意地回答了您的所有相关问题。

研究者或研究者指定的告知医生签名：

*（印刷体）* （手写体） 日期

您与研究者均需签署 2 份相同的知情同意书，双方各保留 1 份。
