# Supplementary figures and images for: Impact of a mobile health intervention based on multi-theory model of health behavior change on self-management in patients with differentiated thyroid cancer: protocol for a randomized controlled trial
Source: Front Public Health. 2024 Jan 11;12:1327442. doi: 10.3389/fpubh.2024.1327442 (PMC10808536; doi:10.3389/fpubh.2024.1327442)

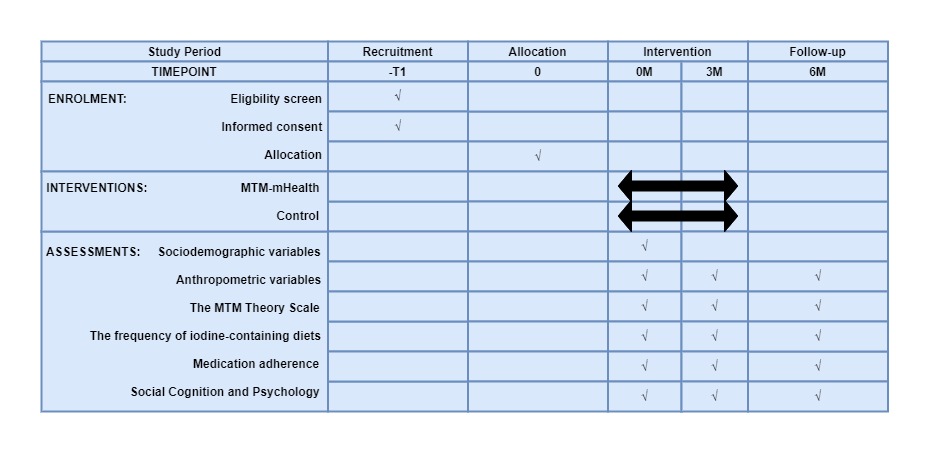

Supplement: Supplementary file 4 [file Image_1.JPEG]
